# Effect of Iron Chloride Addition on Softwood Lignin Nano-Fiber Stabilization and Carbonization

**DOI:** 10.3390/polym16060814

**Published:** 2024-03-14

**Authors:** Maxime Parot, Denis Rodrigue, Tatjana Stevanovic

**Affiliations:** 1 Wood and Forest Science Department, Laval University, Quebec, QC G1V 0A6, Canada; maxime.parot.1@ulaval.ca; 2 Chemical Engineering Department, Laval University, Quebec, QC G1V 0A6, Canada; denis.rodrigue@gch.ulaval.ca

**Keywords:** softwood Organosolv lignin, electrospun lignin fiber, FeCl_3_, thermal oxidative stabilization, carbonization, carbon fibers

## Abstract

This study presents the effect of iron chloride addition on the production of nanocarbon fibers from softwood Organosolv lignin. It was shown that adding 2% FeCl_3_ to the lignin solution before electrospinning to produce lignin nanofibers increased the thermal resistance of lignin fibers during stabilization. FTIR and XPS analyses of the lignin fibers stabilized with and without FeCl_3_ revealed that the temperature rate could be increased in the presence of FeCl_3_ from 1 to 3 °C/min. The optimal temperature to stabilize the lignin fibers was found to be 250 °C, as higher temperatures led to thermal degradation. Also, carbon fibers were successfully produced from pure softwood Organosolv lignin fibers. Carbonization tests were conducted under nitrogen and the best parameters were determined to be a ramp of 10 °C/min until 600 °C with a holding time of 2 h. Furthermore, the effect of 2% FeCl_3_ addition in the lignin solution was investigated during these processes. XPS analysis showed a 93% carbon content for fibers carbonized with and without FeCl_3_ addition, while SEM images revealed some surface roughness in fibers with FeCl_3_ after carbonization. These results confirm that FeCl_3_ addition influences the carbon nanofiber production.

## 1. Introduction

Carbon fibers (CFs) and carbon nanofibers (CNFs) have emerged as a versatile class of materials with several applications spanning diverse fields. CNFs can be used as reinforcing agents in composite materials, enhancing their mechanical properties, such as strength, rigidity, and impact resistance [1,2,3,4]. CNFs are also used in energy storage devices such as batteries and supercapacitors due to their high surface area and electrical conductivity [5,6]. Moreover, CNFs can be functionalized to remove pollutants as their high surface area and porosity make them effective adsorbents for organic contaminants or pollutants [7,8].

Today, most carbon fibers (CFs) are made from polyacrylonitrile (PAN), which is a petroleum-based molecule [9,10]. However, the recent development of bio-based CFs has attracted significant attention due to their sustainable and environmentally friendly attributes [11,12]. Derived from renewable sources such as lignocellulosic biomass, these CFs offer distinct advantages over their non-renewable counterparts, such as the abundance and cost-effectiveness of the feedstock. For example, Warren et al. reported that the production of PAN represented 50% of the CF manufacturing cost [13,14].

Because of its abundance, availability, and renewable character, wood and its derivatives have quickly imposed themselves among the candidates to replace PAN. In particular, lignin as an aromatic biopolymer, has been shown to be more promising than cellulose or hemicelluloses [15,16]. Nevertheless, lignin is a complex copolymer having a random structure depending on the plant origin, growing conditions, and extraction method [17,18]. The extraction conditions can also affect the structure of isolated lignin making its study more complex [19]. These factors represent significant challenges in the transformation of biomass into carbon fiber. The variability in lignin type, quality, and yield represents an obstacle to gathering sufficient data to devise an universally applicable manufacturing process for carbon fiber production using lignins as starting polymers.

Lignin can be transformed into fiber by several processes. Among them, electrospinning is a versatile and innovative technique with several advantages. Its tunability, scalability, and versatility make it a valuable tool to produce nanofibers. This is why this technique was selected in this study to produce lignin fibers at a nanometer scale.

Lignin fiber (LF) is a very brittle material in itself, requiring adequate heat treatment to consolidate and become a carbon fiber. In a first step, thermal oxidative stabilization is required, commonly performed under air to increase the LF glass transition temperature (Tg), or even to eliminate it [15,20]. For this step, the temperature rise is very slow (0.2 to 5 °C/min) to avoid harsh degradation of the lignin structure and allowing for molecular rearrangements [15,21,22]. Finally, a carbonization step makes it possible to eliminate heteroatoms such as oxygen and hydrogen. At the same time, the lignin structure becomes more condensed through an increase in carbon–carbon bonds, leading to the formation of condensed ring structures containing sp^2^ hybridized carbons to produce a carbon fiber with very high tensile strength [23].

One of the biggest challenges in the manufacture of biobased CNFs is to obtain good mechanical properties. Up to now, most of the biosourced NCFs obtained to date do not have sufficient properties to counteract petro-sourced commercial NCFs [24]. Constant innovation is therefore required to find an efficient method of manufacturing biosourced NCFs. This study represents a significant innovation within the field, as no prior investigation has explored the impact of FeCl_3_ on Organosolv lignin during the stages of stabilization and carbonization. Exploring novel approaches is imperative in uncovering manufacturing methods capable of producing bio-based carbon fibers with properties comparable to carbon fiber derived from polyacrylonitrile. Moreover, the efficacy of FeCl_3_ in electrospinning, demonstrated by its ability to reduce fiber diameter and elevate glass transition temperature [25], could underscore its potential in enhancing the properties of lignin-based carbon fibers.

Iron cations are known to interact with lignin moieties. In our previous study, we showed the beneficial effects of adding 2% FeCl_3_ in electrospinning [25]. However, its effect has not been studied so far during the stabilization and carbonization process for carbon fiber manufacturing. This is why, in this study, we evaluated the effect of FeCl_3_ addition on electrospun lignin fibers for both the stabilization and carbonization steps. Also, we produced, for the first time, carbon fibers from pure softwood Organosolv lignin; a lignin substrate otherwise rarely studied for carbon fiber production.

## 2. Materials and Methods

### 2.1. Lignin Fiber

The material used for electrospinning was an Organosolv lignin from black spruce (*Picea mariana*) [26]. This Organosolv lignin was dissolved in dimethyl formamide (DMF) from Sigma-Aldrich, St. Louis, MO, USA (CAS: 68-12-2) at 57 wt.% with (2 wt.%) and without iron chloride III (FeCl_3_) from Sigma-Aldrich, St. Louis, MO, USA. Then, these solutions were electrospun (homemade equipment) without any polymer addition using the following parameters: a flow rate of 0.5 mL/h, a needle-to-collector distance of 20 cm with an electrical tension of 20 kV for 5 min [25]. During electrospinning, the fibers were collected on an aluminum foil and remained on it during the stabilization phase because pure lignin fibers are too fragile to be handled alone.

### 2.2. Stabilization and Carbonization of Electrospun Lignin Fibers

Before stabilization, the lignin fibers were left to dry in air for 24 h to allow for residual DMF to evaporate. To stabilize the lignin fibers, a Lindberg/Blue M muffle furnace from Thermo Scientific (Waltham, MA, USA) was used under air. After stabilization, the lignin fibers were carbonized. For this purpose, an OTF-1200X tube furnace from MTI Corporation (Richmond, CA, USA) was used with a nitrogen purge for 30 min to remove air before heating. Different rates and maximum temperatures were tested as reported in Table 1.

For example, Ox1 corresponds to an oxidative stabilization of lignin fibers from 30 to 200 °C, with a temperature rise of 1 °C/min, and with the maximum temperature maintained for 2 h.

### 2.3. Lignin Fiber Analysis

The lignin fibers were analyzed using Fourier-transform infrared (FTIR) spectroscopy via attenuated total reflectance (ATR) FTIR/FT-MIR (mid-infrared). A Perkin Elmer Spectrum 400 was used at wavenumbers ranging from 4000 to 550 cm^−1^ with 64 scans at a resolution of 4 cm^−1^. To compare the stabilized lignin fiber with a semi-quantitative method, the band at 1596 cm^−1^ was normalized. This band corresponds to aromatic vibration, which is not expected to change during stabilization.

Raman spectra of the carbonized fiber mats were recorded on a SENTERRA II Raman microscope (Bruker Optics Inc., Billerica, MA, USA) equipped with a 532 nm laser. A total of 5 accumulations per 1 s at a 25 mW laser power were collected in the range 50−4000 cm^−1^ using a ×20 microscope objective to determine carbon hybridization.

Thermogravimetric analysis was performed with a TGA/SDTA851 (Mettler Toledo, Columbus, OH, USA) under a nitrogen atmosphere (50 mL/min) from 35 to 850 °C at a heating rate of 10 °C/min and under air (50 mL/min) from 35 to 500 °C at a heating rate of 10 °C/min. The derivative of the TGA curve (DTG) was also analyzed.

X-ray photoelectron spectroscopy (XPS) (Physical Electronics, Chanhassen, MN, USA) was used for surface composition elemental analyses.

An optical microscope Keyence VHX-7000 and a scanning electron microscopy (SEM) FEI Quanta 250 at 7.5 kV and 4 spots with a secondary electron mode, were used for morphological analysis. Three images were taken randomly for each sample, and the fiber diameter distribution was determined by measuring the diameter of all the fibers intersecting the median horizontal axis of each image (at least 80 random fiber diameters were measured for each sample). Image analysis was performed using the ImageJ software (Version 1.53e).

## 3. Results and Discussion

### 3.1. Effect of FeCl_3_ Addition on Lignin Fiber Stabilization

The thermal stability of the raw material plays a pivotal role in carbon fiber manufacturing. Insufficient stability can lead to material combustion or melting during the oxidative stabilization process, presenting a significant challenge in carbon fiber production. Furthermore, it is noteworthy that softwood lignin exhibits a higher degree of condensation compared to hardwood lignin. This structural attribute, characterized by a branched and crosslinked configuration, contributes to enhanced stabilization properties by mitigating the risk of material melting [20]. Also, as reported in our previous study, the addition of 2 wt.% of FeCl_3_ is beneficial for the thermal stability of lignin fibers [25]. The results of all characterizations have been reported in that study. DSC analyses showed an increase in Tg for lignin fibers stabilized with ferric chloride.

**Figure 1:**
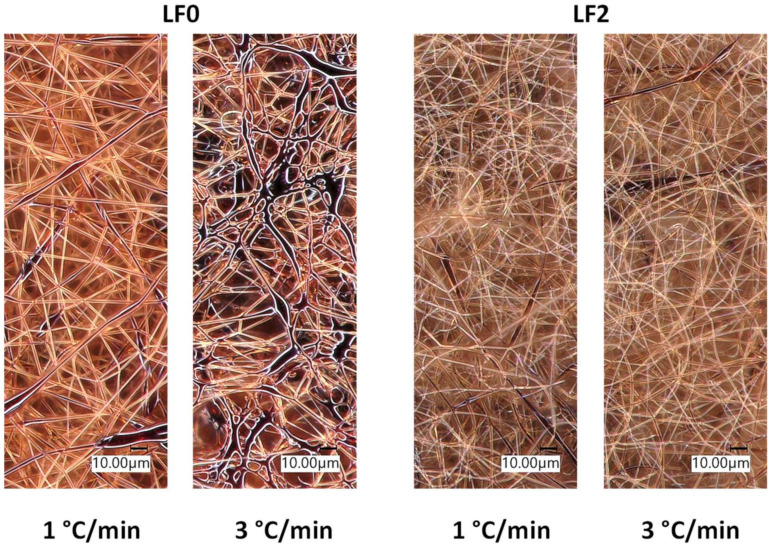
Optical microscopy images of lignin fibers after stabilization at 200 °C.

Both lignin fibers with (LF2) and without (LF0) FeCl_3_ were tested for stabilization. The morphology observed via optical microscopy shows that the lignin fibers with FeCl_3_ are more stable than those without (Figure 1). At a heating rate of 1 °C/min from 30 to 200, 250 and 300 °C, both fibers (LF2 and LF0) keep their shape. But for faster rates, LF0 melts while LF2 keeps its shape. At 2 °C/min and above, LF0 fibers melt while LF2 fibers remain intact. An example is shown in Figure 1, where FeCl_3_-free fibers can be seen intact after stabilization heating rate at 1 °C/min, while the same fibers after stabilization at 3 °C/min have fused. Figure 1 also shows that LF2 fibers do not fuse at 3 °C/min, unlike LF0 fibers. These results show that the addition of FeCl_3_ makes it possible to improve the thermal resistance of lignin fibers. However, even at 1 °C/min, but until 400 °C, both fibers were totally burned and only ashes remained.

The lack of literature on the effect of FeCl_3_ during lignin fiber stabilization makes the understanding of the intrinsic mechanisms difficult. In order to better understand the interactions between iron and lignin during this phase, several spectroscopic analyses were carried out.

**Figure 2:**
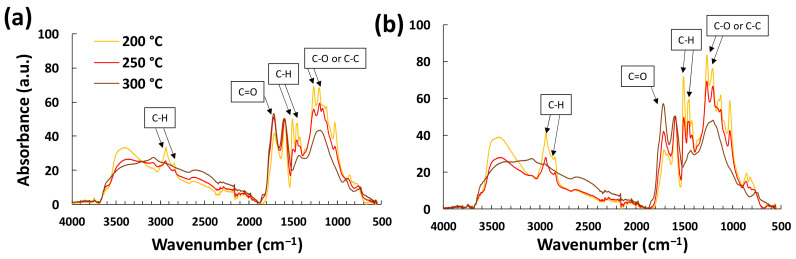
FTIR spectrum of lignin fibers stabilized at 200, 250, and 300 °C: (**a**) LF0 and (**b**) LF2.

The FTIR analysis shows that, for the same maximum temperature and regardless of the presence of ferric chloride (LF0 and LF2), the molecular structures after stabilization are similar after heating at all rates (1, 2 and 3 °C/min). However, the maximum temperature has an influence on the molecular structure as shown in Figure 2.

The FTIR analysis shows slight differences between the spectra of LF0 and LF2. The intensities of the infrared bands of LF0 vary in the same way (a decrease or increase in the same bands), but less with increasing temperature compared to the bands of LF2. The band at 3500 cm^−1^ is attributed to hydroxyl functions and decreases with increasing temperature. All the bands at 2930, 2840, 1454, and 1425 cm^−1^, which are associated with the C–H of different functions, also decrease with increased temperature [27,28]. For comparison, all the spectra were normalized with the 1596 cm^−1^ band because it represents the aromatic rings which should not change with stabilization. The spectra seem to indicate that hydrogen left the lignin structure (oxidation process) as indicated by the less intense bands related to H atom vibrations, while the increased oxidation degree is also indicated by more intense C=O band at 1710 cm^−1^, associated with C=O unconjugated carbonyl groups [20]. The peaks at 1265 and 1204 cm^−1^ correspond to conjugated C–O or C–C vibrations, which appear to have merged into a single broad band at 1250 cm^−1^ when treated at 300 °C. This indicates that C=O carbonyls were generated.

Overall, the spectra show that the C=O bands are more intense for samples stabilized at 250 °C than for those stabilized at 200 °C, while the samples stabilized at 300 °C are reduced to only five broad bands. This suggests that some degradation occurred, as the remaining bands correspond to the strongest bonds (C–C or C=O for example) and the weaker bonds (such as ether or hydroxyl bonds) disappeared. These results also indicate a beginning of combustion which will be confirmed via TGA under air. These analyses provide very interesting information because, if the morphology of LF2 seems to remain intact under optical microscopy, it turns out that the molecular structure remained the same for both samples (i.e., with or without FeCl_3_).

**Figure 3:**
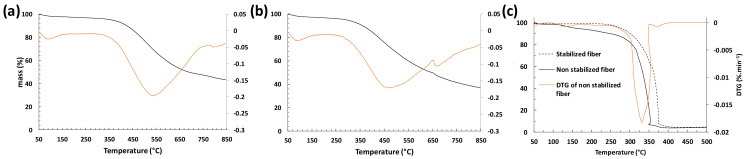
TGA and DTG curves under nitrogen after oxidation at 200 °C at 1 °C/min for: (**a**) LF0, (**b**) LF2, and (**c**) stabilized and non-stabilized fiber under air.

This thermal degradation hypothesis is supported by the TGA results presented in Figure 3, showing that the degradation started only above 250 °C.

As mentioned earlier, thermal treatment under air at 400 °C and 1 °C/min completely decomposed the lignin fibers and only ashes remained. To confirm this phenomenon, TGA under air was performed and the results of Figure 3c show that the degradation of lignin occurred from 300 °C and was almost complete around 400 °C, indicating that combustion occurred. These results also show that combustion occurs for both fibers (stabilized or not) which confirms the importance of the carbonization step under nitrogen.

To better understand the behavior of material during stabilization, XPS analyses were performed. Table 2 shows that the elemental composition of both fibers remained the same after all stabilization conditions; i.e., about 25.8 ± 0.9% oxygen and 74.2 ± 0.9% carbon (hydrogen could not be analyzed). However, before stabilization, the fibers are composed of 79.5 ± 0.5% carbon and 20.5 ± 0.5% oxygen. Ding et al. observed an increase in the oxygen content of around 3% [22], while Cho et al. obtained a 2% increase after stabilization at 250 °C [27], which is lower than (but close to) the 5% oxygen difference reported in Table 2.

**Figure 4:**
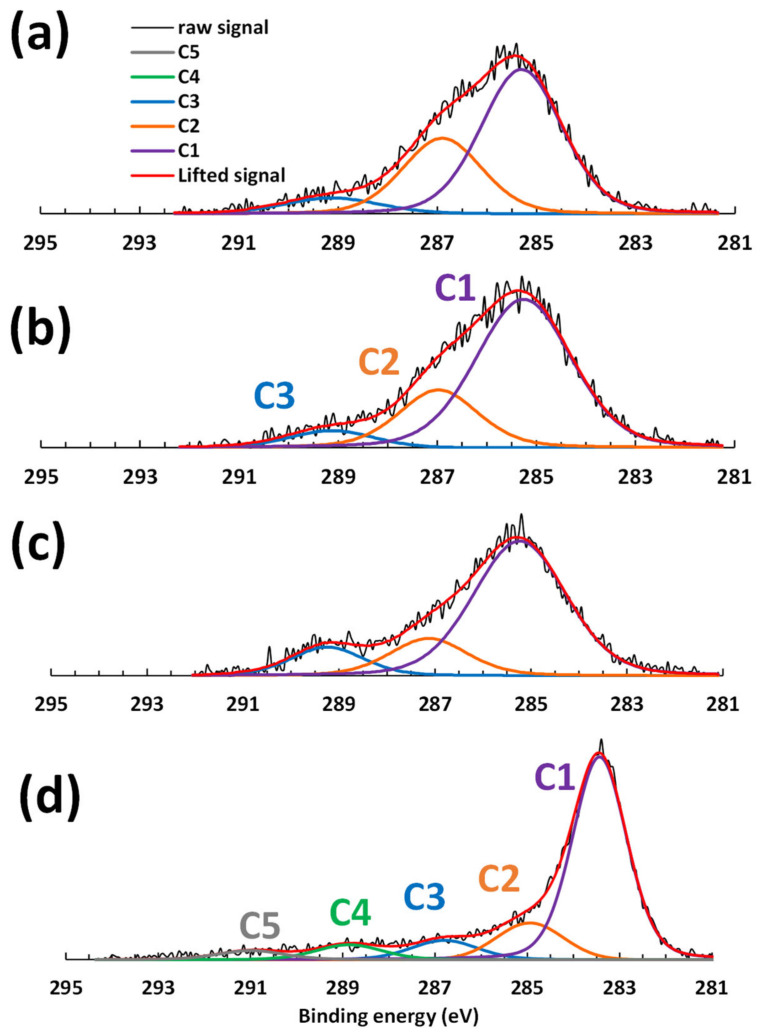
High-resolution XPS spectra of LF2 stabilized at 1 °C/min up to: (**a**) 200 °C; (**b**) 250 °C; (**c**) 300 °C and (**d**) carbonized fibers.

A difference can be seen in the peaks on the high resolution XPS analyses. Figure 4a–c report that the spectrum deconvolution includes three types of carbons. C3 has a binding energy of around 289 eV, which reflects highly oxidized carbons linked to oxygens. C2 is around 287 eV and reflects an alcoholic C–O bond, while C1 is linked to C–C, C–H, or C=C [15,29]. At 1 °C/min, C3 and C1 increase with increasing temperature for LF0 and LF2, reflecting oxidation. The areas of the C3 peaks change from around 6, 7, and 10 for oxidation at maximal temperatures of 200, 250, and 300 °C, respectively (Ox1, Ox2, and Ox3), while the areas of the C1 peaks change from 44, 47, and 49, respectively.

A decrease in C2 is in agreement with an increase in C3 with a substantial decrease at 300 °C. The areas change from 25 and 22 to 13 at 200, 250, and 300 °C, respectively. These results reflect an increase in carbon oxidation as suggested by Brodin et al. and Braun et al. who mentioned the direct oxidation of phenolic groups and hydroxymethyl groups during stabilization [15,29]. In fact, the loss of hydrogen (by H_2_ or H_2_O formation) can lead to carbon–carbon or carbon–oxygen bonds, leading to an increase in the oxidation degree of the lignin polymer. The peak areas are similar between LF0 and LF2 indicating that FeCl_3_ does not influence the oxidation. Moreover, the XPS results do not show any significant differences between the samples produced at different temperature ramps.

The XPS results show an increase in the carbon–oxygen double bonds (C=O) with an increase in oxygen content, which indicates that lignin oxidation occurred and confirms the FTIR results. This increase could be explained by the elimination of hydrogen via water or H_2_ molecule formation. Moreover, optical microscopy (Figure 1) shows the effect of heating rate because a high value melts the fibers, except for fibers containing FeCl_3_. These results are in agreement with the literature mentioning that the temperature ramp is a crucial factor for oxidation [29]. The results are also in agreement with MacDermind-Watts et al. who observed a better thermal stability for their samples with FeCl_3_ [30].

While the intricate complexation mechanism between iron cations and lignin phenolic hydroxyls remains complex, our results suggest that FeCl_3_ plays a significant role in enhancing the thermal stability of lignin fibers, thereby rendering it beneficial for carbon fiber application. One hypothesis that could be proposed is by the interaction between the iron cations with the free radicals generated during the heat treatment of lignin. The C–O and C–H linkages would be easier to break, and carbon–carbon bonds would be preferably formed due to iron complex formation.

In the literature, most stabilizations were performed at temperature rates below 1 °C/min. This difference may be due to the softwood lignin being basically more condensed than hardwood lignin [20,31]. Moreover, the addition of FeCl_3_ has never before been tested in stabilization for CF application and this addition helps to increase the temperature ramp which is much higher than the ramps reported so far. So, FeCl_3_ addition makes it possible to reduce the thermal treatment time for stabilization.

### 3.2. Effect of FeCl_3_ Addition on Lignin Fiber Carbonization

LF0 and LF2 were stabilized at 1 °C/min (Ox2 in Table 1). The stabilized fibers led to pyrolysis for Ca1 and Ca2 under nitrogen (nothing remains after treatment). This indicates that 1 and 5 °C/min are too slow. Only 10 °C/min was suitable for carbon fiber production, which is faster than in most studies in the literature [31]. These results are unexpected, since a slower temperature increase should be favorable to good carbonization, as molecular bonds would have more time to rearrange, whereas too rapid an increase in temperature generally leads to thermal degradation. One hypothesis could be that the activating properties of iron lead to material degradation if the heating time is too long, but this would not explain the occurrence of the same phenomena for lignin nanofibers without iron.

**Figure 5:**
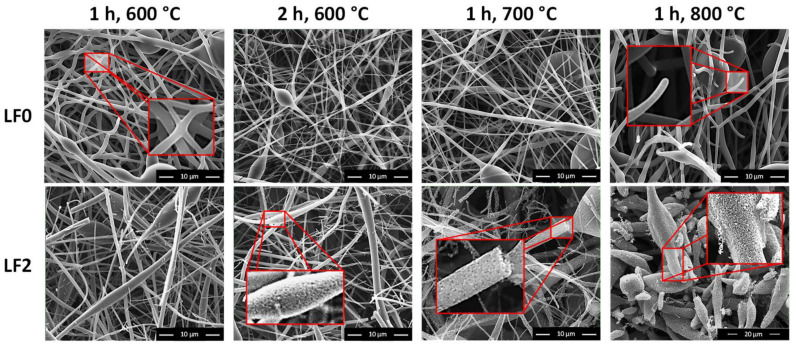
SEM images of the resulting carbon fibers.

Figure 5 shows that LF0 after carbonization under the conditions Ca3, Ca4, and Ca5 show a smooth structure without roughness with a slight fusion of the fibers at specific location as we can see on zooms in the red shapes for LF0. This fusion could provide better mechanical resistance and electrochemical properties according to the literature [31,32].

Moreover, these results confirm the possibility of producing carbon fibers from pure softwood Organosolv lignin as all the carbon fibers made from lignin fibers so far were made from Kraft or various hardwood lignins [33]. On the other hand, LF2 shows surface roughness after carbonization that increases with increasing temperature as we can see on zooms in the red shapes for LF2 (Figure 5). This roughness is the result of the iron’s chemical activation effect [30]. Figure 5 shows that the maximum temperatures of 600 °C and 700 °C are adequate to keep the fibers’ shape, but 800 °C is too high since the fibers become thermally degraded for LF0 or transformed into porous particles for LF2. For the latter, a foaming phenomenon seems to occur due to the large diameter and porous structure of the remaining particles. Finally, 900 °C is too high since the fibers become pyrolyzed after heating due to their decomposition into volatiles such as CO or CO_2_ [34].

In the literature, different carbonization conditions are reported depending on the lignin used. For example, Cao et al. carbonized a blend of cellulose acetate and poplar lignin at 4 °C/min until 600 °C maintained for 2 h [35], while Svinterikos et al. used a blend of Kraft lignin with PET, carbonized at 5 °C/min for 1 h and Schreider et al. used a blend of hardwood Organosolv lignin cellulose acetate electrospun and carbonized at 2 °C/min for 1 h [36,37].

#### 3.2.1. CF Diameter

**Figure 6:**
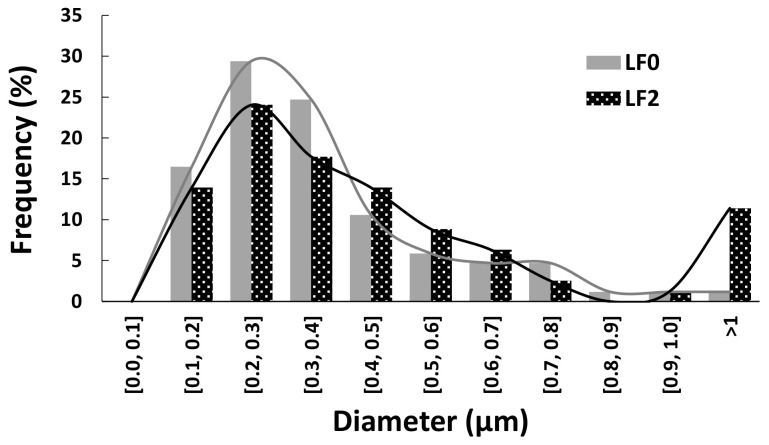
Size distribution of the carbon fibers.

Carbon fibers diameters were calculated for fibers carbonized at 600 °C during 1 h for LF0 and LF2. The size distribution is shown in Figure 6.

Figure 6 shows that the carbon fiber diameters are mainly in the range of 0.1–0.6 µm and these values correspond to those reported in the literature which vary substantially from 0.2 to 2 µm for electrospun fibers produced from lignin [19,38]. The difference in fiber size is due to the electrospinning process, which generates fibers of slightly varying diameter. As reported by Ruiz-Rosas et al. (2010)*,* the fiber diameter decreases by 40% on average during transformation into CF, which is the case here as the values decreased from 0.2–1 µm after electrospinning to 0.1–0.6 µm after carbonization. This reduction is mainly due to the release of CO, CO_2_, and H_2_O molecules while creating new carbon-carbon bonds, which condense the material, reducing the diameter [34]. However, there is no significant difference between the diameter of carbon fiber from LF0 and LF2.

#### 3.2.2. Surface Analysis of Carbon Fibers

To determine the elemental composition of the carbon fibers obtained at 600 and 700 °C, they were analyzed by XPS and the results are reported in Table 2. Fibers with and without FeCl_3_ gave similar results, so only the averages of both are reported in Table 2. Iron (Fe) could not be detected because it forms complexes with lignin as reported in a previous study [25], and is therefore not located on the surface. Since XPS is a surface analysis, only carbon and oxygen were analyzed. Within experimental uncertainty, the elemental analysis shows the same atomic composition for LF0 and LF2 after carbonization. However, differences are observed between the carbonization conditions. At 700 °C for 1 h (Ca5), the carbon content reached 95.3 ± 0.9%, while at 600 °C for 1 h (Ca3) and for 2 h (Ca4), the carbon content reached 93.0 ± 0.8% on average. Moreover, increasing the time at 600 °C from 1 h to 2 h did not significantly change the carbonization rate.

Ding et al. produced CF with a mixture of lignin and PAN carbonized at 5 °C/min up to 1000 °C maintained for 30 min to obtain CF with only 92% carbon [22]. This result shows that, in contrast to pyrolysis [39], heating at a higher temperature may not be necessary to increase the carbon content, as higher values were obtained in our case using only 600 °C.

High resolution XPS has shown that the rate of sp^2^ carbon increased during carbonization, as seen on Figure 4d. All the bands are shifted by about 2 eV, which indicates a loss of oxygen. Also, according to Kaciulis [40], the new band C1 at 283 eV represents sp^2^ carbon, C2 at 285 eV represents sp^3^ carbon, C3 at 287 eV represents hydroxyls, C4 at 289 represents carbonyls, and C5 at 291 eV represents carboxyls. So, the carbonization treatment increases the sp^2^ carbon content.

The SEM and XPS results show that carbonization at 600 °C maintained for 1 h for both lignin fibers (LF0 and LF2) increased the carbon content from 74% to 93%, while 700 °C further increased the value up to 95% for both samples. Nevertheless, LF0 gave smooth and slightly melted fibers, while those of LF2 were more porous due to the presence of iron. These results show that the addition of iron leads to the formation of pores in the final CF structure after carbonization, which can be very interesting depending on the final application.

#### 3.2.3. Structural Order Characterization

**Figure 7:**
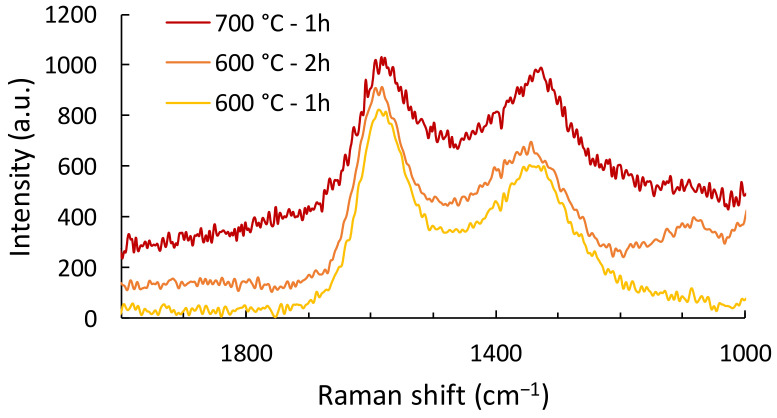
Raman spectra for LF0 after carbonization.

Raman spectroscopy is a widely used technique to determine the degree of structural order of carbons in carbon fibers. For this purpose, the two peaks used are those corresponding to sp^3^ carbons, known as disordered/amorphous, corresponding to carbons in pure diamond (called D peak, centered around 1330 cm^−1^) and sp^2^ carbons, known as ordered, corresponding to graphite (called G peak, centered around 1580 cm^−1^). The ratio of peak intensities (R = I_D_/I_G_) makes it possible to compare the order of the carbon structure with those from the literature. Figure 7 presents the Raman spectra for LF0 only, knowing that the spectrum for LF2 was similar.

Firstly, the I_D_/I_G_ ratio is similar for LF0 and LF2. The fibers after Ca3 (600 °C for 1 h) show a ratio of 0.81 ± 0.06, while the ratio is 0.93 ± 0.09 after Ca5 (700 °C for 1 h). The literature results reported so far seem to indicate that the lower this ratio, the more sp^2^ hybridized carbons are present and, therefore, the higher the degree of structured carbon [41]. So, Raman analysis shows that there is no difference in the carbon hybridization between LF0 and LF2, and that 700 °C decreased the ordered structure of carbon compared to 600 °C. Moreover, the ratio after Ca4 is 0.75 ± 0.02, which indicates that keeping the fibers at 600 °C for 2 h makes it possible to increase the sp^2^ hybridization compared to heating at the same temperature for 1 h.

Shi et al. obtained R of 0.97, 0.92, and 0.85 for CF from different Organosolv lignins [19]. Wang et al. showed that the addition of nitrogen led to more disordered carbon as R increases from 1.32 to 1.51 upon that addition [32]. Cho et al. showed that the addition of crystalline nanocellulose increased the disorder of carbon fibers from 1.4 to 1.7 after stabilization. Without stabilization, the ratio increased up to 2.5 [21]. Cao et al. also reported a ratio between 0.78 and 0.72 for CF produced from poplar lignin (with polymer addition), which is close to our results [35].

This study has several limitations, mainly caused by the brittleness of the fibers obtained. This property was not improved upon the addition of FeCl_3_ which made mechanical analyses impossible. In addition, FeCl_3_ content could not be quantified in the stabilized and carbonized fiber since the measurements were not sufficiently accurate for this purpose (SEM-EDX, XRD). However, it has been possible to evaluate in this study the effect of FeCl_3_ in the stabilization and carbonization stages.

## 4. Conclusions

The results obtained in this study have contributed to understanding of the effect of adding iron III chloride (FeCl_3_) to lignin fibers before the stabilization and carbonization stages of carbon fiber fabrication. During stabilization, the fibers with 2% FeCl_3_ showed better thermal resistance. FTIR and TGA analyses on the stabilized fibers showed an onset of thermal degradation around 300 °C, which made it possible to determine that the best stabilization temperature is around 250 °C for lignin with and without FeCl_3_. However, the addition of FeCl_3_ enabled a substantial increase in the achievable heating rate, elevating it from 1 to 3 °C/min while preventing the melting of the fibers with FeCl_3_. This advancement would make the fabrication process more efficient and less energy intensive. Carbonization tests and Raman spectroscopy showed that the best carbonization parameters determined for softwood (black spruce) Organosolv lignin were 10 °C/min under nitrogen, until 600 °C for 2 h, as these conditions seem to favor the sp^2^ hybridization of carbons for fibers both with and without iron chloride addition.

SEM analysis showed that 2% of FeCl_3_ generated roughness in the carbon fibers but kept fibers infusible, while pure lignin fibers (produced without FeCl_3_) were carbonized, producing smooth but slightly crosslinked fibers.

## Figures and Tables

**Table 1:** Experimental conditions for lignin fiber stabilization and carbonization.

Code	Rate (°C/min)	Initial Temp. (°C)	Final Temp. (°C)	Holding Time (h)
Stabilization				
Ox1	1	30	200	2
Ox2	1	30	250	2
Ox3	1	30	300	2
Ox4	1	30	400	2
Ox5	2	30	200	2
Ox6	2	30	250	2
Ox7	2	30	300	2
Ox8	3	30	200	2
Ox9	3	30	250	2
Ox10	3	30	300	2
Carbonization				
Ca1	1	R.T.	500	1
Ca2	5	R.T.	700	1
Ca3	10	R.T.	600	1
Ca4	10	R.T.	600	2
Ca5	10	R.T.	700	1
Ca6	10	R.T.	800	1
Ca7	10	R.T.	900	1

**Table 2:** XPS composition of lignin fibers (LFs) and carbon fibers (CFs). The atomic composition is similar for LF0 and LF2.

Sample	LF	LF after Oxidation	CF after Ca3	CF after Ca4	CF after Ca5
Carbon (%)	79.5 ± 0.5	74.2 ± 0.9	93.3 ± 0.6	92.5 ± 0.8	95.3 ± 0.9
Oxygen (%)	20.5 ± 0.5	25.8 ± 0.9	6.7 ± 0.6	7.5 ± 0.8	4.7 ± 0.9

## Data Availability

Data are contained within the article.
